# Assessment of the Dimensions of Coronary Arteries for the Manifestation of Coronary Artery Disease

**DOI:** 10.7759/cureus.46606

**Published:** 2023-10-06

**Authors:** Muhammad Muneeb, Nasia Nuzhat, Attaullah Khan Niazi, Ammar H Khan, Zanib Chatha, Tahseen Kazmi, Saira Farhat

**Affiliations:** 1 Interventional Cardiology, Shalamar Medical and Dental College, Lahore, PAK; 2 Applied Physics, University of Engineering and Technology, Lahore, PAK; 3 Cardiac Surgery, King Edward Medical University, Lahore, PAK; 4 Cardiovascular Surgery, Imran Idrees Hospital, Sialkot, PAK; 5 Community Medicine, Central Park Medical College, Lahore, PAK

**Keywords:** coronary artery diameter, left circumflex artery, left anterior descending, left main stem, right coronary artery

## Abstract

Introduction: The size of the coronary artery influences the effective outcome of therapeutic measures like coronary artery bypass graft (CABG) surgery, percutaneous coronary interventions (PCI), and diagnosis of coronary artery disease. Patients' age, gender, BMI, anatomical variations, and increased left ventricular size all have an effect on coronary artery parameters.

Objective: This study aims to compare the average size of the coronary arteries of the Pakistani population in both sexes for manifestation of coronary artery disease.

Methodology: For the analysis of the coronary arteries, 100 patients of both sexes, male and female, were taken. X-ray angiography was performed for two-dimensional images of coronary arteries. For diameter measurement, images were visualized on quantitative coronary angiography (QCA) in different views (caudal and cranial views). The diameters of the left main coronary artery (left main stem/LMS), left anterior descending (LAD), left circumflex (LCx), and right coronary artery (RCA) were measured on angiograms. Data about the dimensions of the coronary artery was gathered through quantitative angiography. Data analysis was done through SPSS version 26 (IBM Corp., Armonk, NY).

Results: There is a notable distinction in the average diameters among the proximal LAD (3.12), mid-LAD (2.40), and distal LAD (1.29). A statistically significant difference is evident among mid-LCx, distal LCx, and proximal LCx (p-value < 0.001). Likewise, the average diameter of the distal RCA (1.89) was smaller when compared to the mid-RCA (3.19) and proximal RCA (3.78). However, there was no significant difference in the average diameter among mid-LMS, distal LMS, and proximal LMS (p-value = 0.09).

Conclusion: The average diameter of distal RCA was smaller when compared to mid-RCA and proximal RCA. The average size of proximal LAD and proximal LCx was comparatively larger than mid- and distal LAD and LCx. The findings of current research will be beneficial for the diagnosis and management of coronary artery disease patients.

## Introduction

Although significant progress has been made in the detection and treatment of coronary artery disease (CAD), it still remains a significant and prominent cause of infirmity and death in both the developing and industrialized worlds [1-3]. Globally, it ranked as the third major cause of death responsible for approximately 17.9 million deaths in a calendar year ranking at 32% of global mortality [2,4,5]. It has been observed that the mortality rate due to CAD in high-income countries has declined, whereas high- and low-income countries bear three-fourth of overall burden of CAD. In South Asia, the prevalence was 50%-300% higher as compared to the other parts of the world [6-8].

Previous studies have shown that the risk of CAD is associated with coronary artery diameter. Such associations can be used to avoid deaths by understanding coronary anatomy, tomography, coronary angiography, and other interventions [9-12]. The heart receives oxygen-rich blood through the right and left coronary arteries. The right and left coronary arteries (RCA and LCA) are further subdivided into smaller arteries. Right posterior descending artery (RPDA) and acute marginal artery (AMA) are the sub-branches of RCA, whereas LCA further divides into left circumflex (LCx) and left anterior descending artery (LAD), and the diameter of the LCA is larger than RCA [13,14].

CAD is an inflammatory disease of the arterial lining of the coronary arteries. The contributing factors to the malfunctioning of the lining are inflammatory response, shear stress of blood flow, oxidative injury, harmful stimuli of vasculature, and factors of vascular dysfunction such as change in blood flow, arterial stiffness, and vascular anatomical changes [15,16]. The diameter of the focused artery such as the coronary artery can be a predictor of CAD. In Asians, the association of CAD with coronary artery diameter is inverse. Patients with a small coronary artery diameter had higher odds of CAD [17].

In addition to this, the literature supports certain other characteristics and considers them to be potential risk factors for CAD. These characteristics include the age of patients, BMI, gender, and race. Coronary artery dimensions were found to be largely impacted by these demographic factors [18-20]. Similarly, other comorbid conditions like hypertension, diabetes, and hyperlipidemia were found to be associated with CAD. Smoking was found significant in the narrowing of coronary artery diameter [21-23]. This investigation aims to evaluate the mean diameter of RCA, LCx, LMS, and LAD in the mid, distal, and radial arteries in the Pakistani population.

## Materials and methods

The study included 100 patients of both sexes, male and female, who were referred for angiography for the diagnosis of arterial disease and were categorized as normal patients. For diameter measurement, images were visualized on QCA (quantitative coronary angiography) in different views (caudal and cranial views). The diameters of the left main coronary artery (left main stem/LMS), LAD, left circumflex (LCx), and right coronary artery (RCA) were measured on angiograms. Data about the dimensions of the coronary artery was gathered at the Shalamar Hospital, Lahore, Pakistan, from December 2021 to June 2022. The OpenEpi sample size calculator was used for the sample size calculation, considering a 90% confidence interval and 80% power of the test. It was based on a mean difference in diameter between the proximal LAD (3.69 ± 0.64) and LCx (3.37 ± 0.63) arteries.

The study protocol was approved by the Institutional Review Board of Shalamar Medical and Dental College (Letter No.: SMDC-IRB/AI/67/2020). Consent was taken from the patients before the collection of data. Data was collected through a structured questionnaire for coronary artery diameter. SPSS version 25 (IBM Corp., Armonk, NY) was used for data analysis. One-way analysis of variance (ANOVA) was used to compare the mean diameter of the main coronary arteries.

## Results

**Figure 1:**
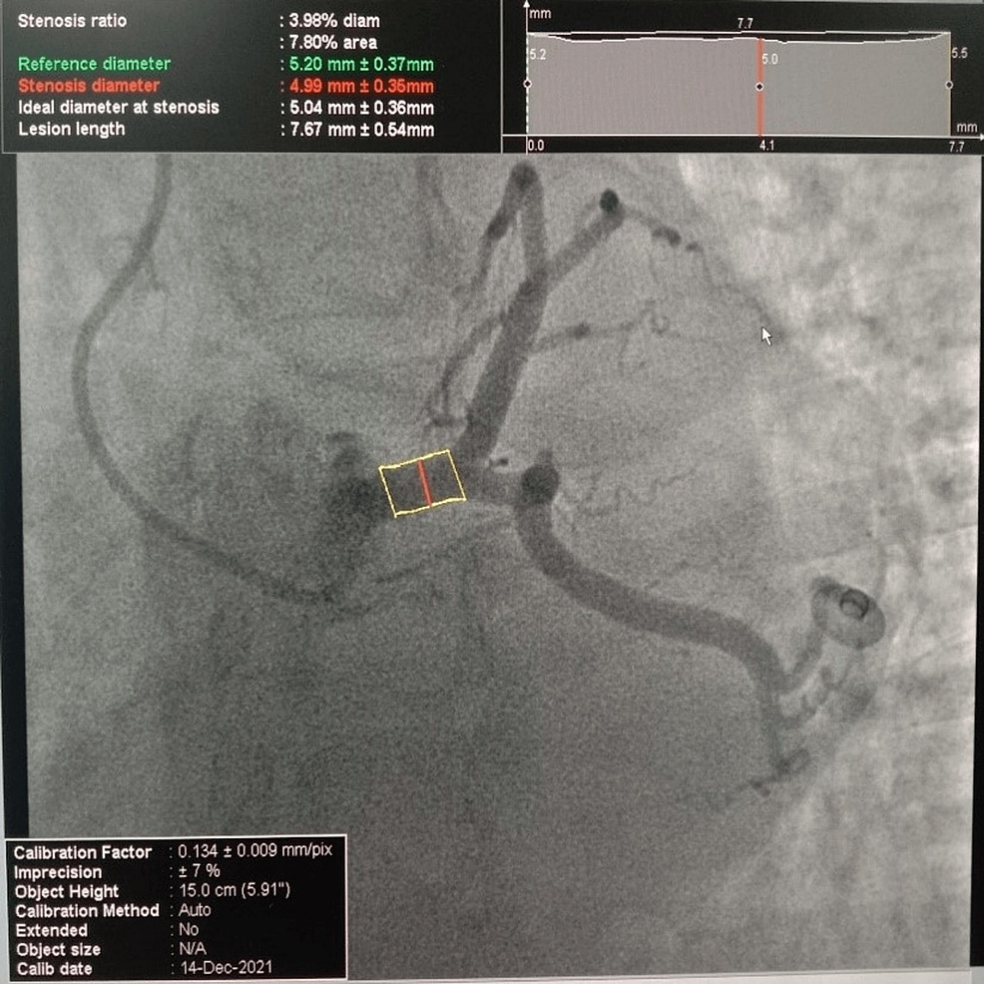
Diameter of left main stem coronary artery measured on quantitative coronary angiography (QCA) at the Shalamar Hospital, Lahore, Pakistan

**Figure 2:**
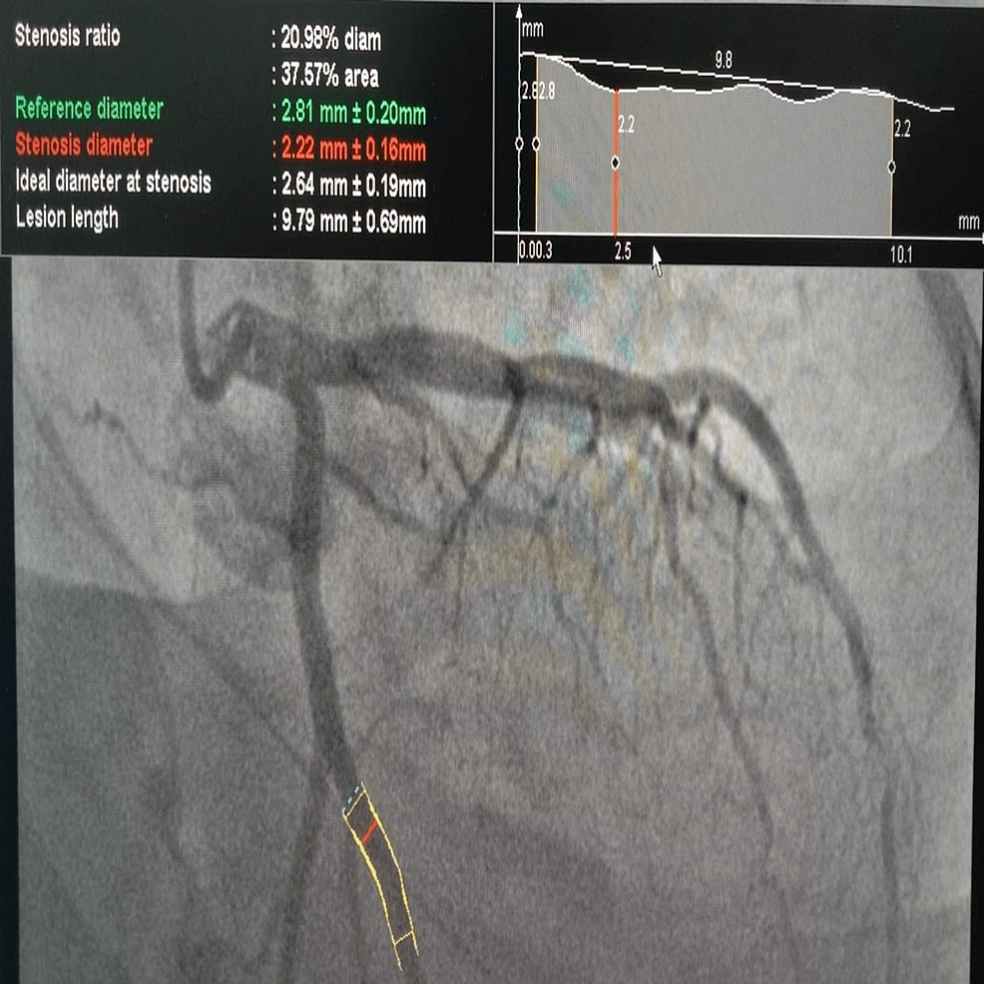
Diameter of left circumflex (LCx) measured on quantitative coronary angiography (QCA) at the Shalamar Hospital, Lahore, Pakistan

Angiography was performed on 100 patients, with an average age of 51.10 ± 11.95 years. Within the complete sample, approximately 49% were female, while the remaining 51% were male. Three left main stem (LMS) segments were evaluated, proximal LMS, mid-LMS, and distal LMS as illustrated in Figure 1. Similarly, in Figure 2 we measured three segments of the LAD, the LAD mid-segment (between the first diagonal and the first septal), the LAD proximal segment (prior to the first septal branch), and the LAD distal segment (after the diagonal branch of the LAD).

**Figure 3:**
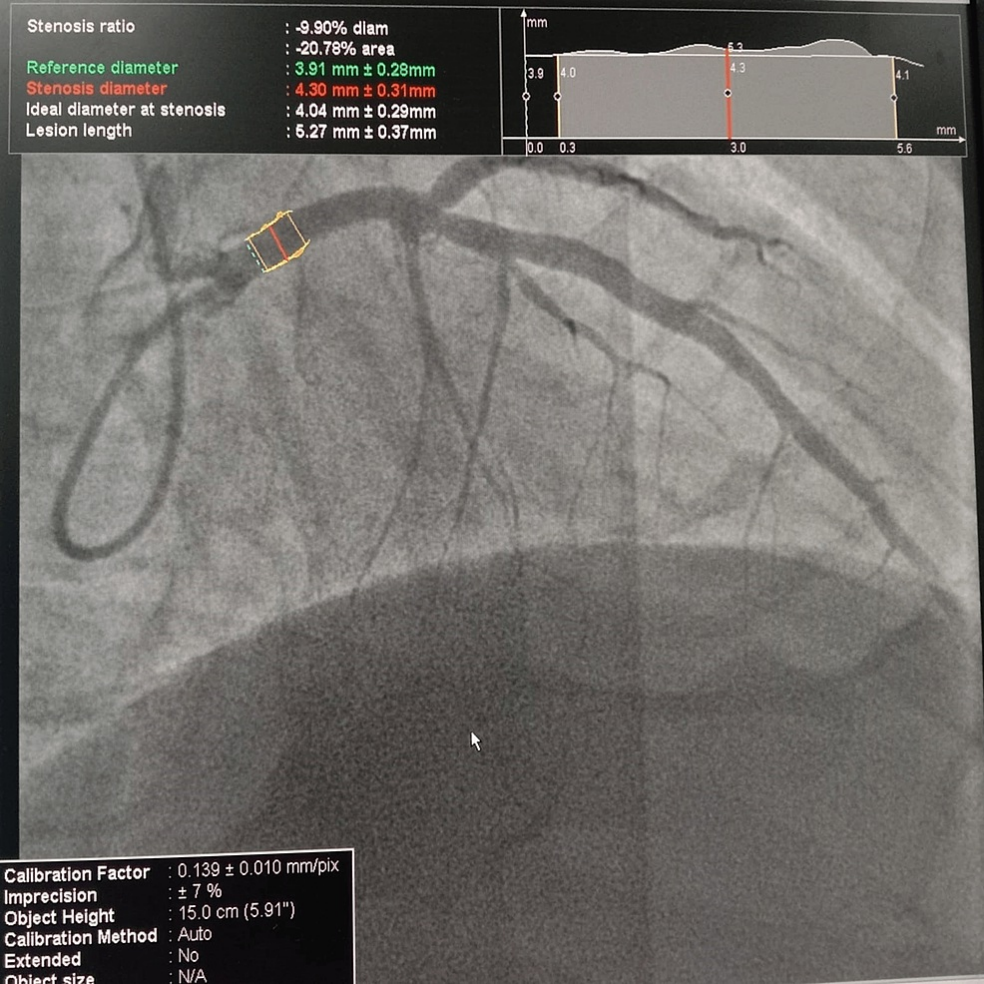
Diameter of left anterior descending (LAD) measured on quantitative coronary angiography (QCA) at the Shalamar Hospital, Lahore, Pakistan

**Figure 4:**
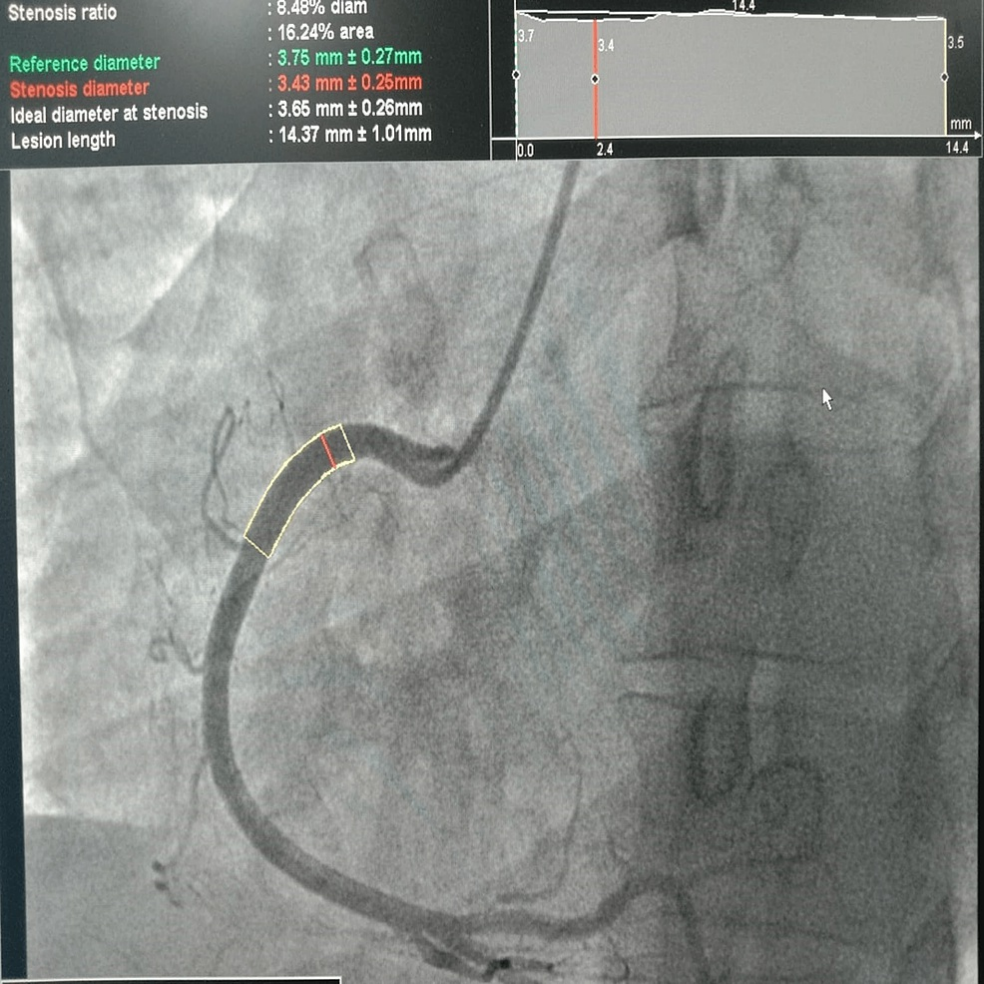
Diameter of right coronary artery (RCA) measured on quantitative coronary angiography (QCA) at the Shalamar Hospital, Lahore, Pakistan

Likewise, Figure 3 shows the three segments of the left circumflex coronary artery LCx, the mid-LCx (adjacent to the first obtuse marginal branch), the distal LCx (beyond the origin of the obtuse marginal branch), and the proximal LCx (before the obtuse marginal branch). Moreover, the RCA was divided into three sections: proximal RCA, mid-RCA, and distal RCA, and we determined the maximum diameter of the posterior descending branch in each of these segments (Figure 4).

The mean diameter and standard deviation of the main coronary arteries and their associated subcategories are shown in Table 1. One-way ANOVA was used to assess the significant difference among the categories of coronary artery. The average diameter of the mid-LAD and distal LAD differed significantly (p-value < 0.001). Furthermore, within the left circumflex region, a noticeable disparity was evident in the average values between LCx (2.86) and distal LCx (1.31), yielding statistically significant results (p-value < 0.001). However, no substantial difference was observed among proximal LMS, mid-LMS, and distal LMS, indicating statistically insignificant results (p-value = 0.09) (Table 1).

**Table 1:** The mean diameter and standard deviation of three segments of main coronary arteries (n = 100) p-value ≤ 0.001 is statistically significant. * F-test (ANOVA). ANOVA: Analysis of variance.

Artery	Sub-divisions	Mean (SD in mm)	P-value
Left main stem (LMS)	Proximal	4.14 ± 0.56	0.09^*^
	Mid	4.03 ± 0.65	
	Distal	4.16 ± 0.74	
Left anterior descending (LAD)	Proximal	3.12 ± 0.62	<0.001^*^
	Mid	2.40 ± 0.37	
	Distal	1.29 ± 0.39	
Left circumflex (LCx)	Proximal	2.86 ± 0.65	<0.001^*^
	Mid	2.49 ± 0.66	
	Distal	1.31 ± 0.44	
Right coronary artery (RCA)	Proximal	3.78 ± 0.64	<0.001^*^
	Mid	3.19 ± 0.60	
	Distal	1.89 ± 0.54	

The distal LCx and RCA had a significantly smaller average diameter than the proximal LCx and RCA as well as the mid-LCx and RCA. At a significance level of 5%, we also examined the mean differences among mid-arteries, distal arteries, and proximal arteries. A significant disparity was detected in the average dimeters of LMS, LAD, LCx, and RCA (p-value < 0.001) (Table 2).

**Table 2:** Average differences in the diameter of main coronary arteries (n = 100) p-value ≤ 0.001 is statistically significant. * F-test (ANOVA). ANOVA: Analysis of variance.

Artery	Proximal artery		Mid-artery		Distal artery	
	Mean	P-value	Mean	P-value	Mean	P-value
Left main stem (LMS)	4.14	<0.001*	4.03	<0.001*	4.16	<0.001*
Left anterior descending (LAD)	3.12		2.40		1.29	
Left circumflex (LCx)	2.86		2.49		1.31	
Right coronary artery (RCA)	3.78		3.19		1.89	

## Discussion

The present study provides insights into the diameter of RCA and sub-branches of LCA among 100 patients. Measurements of diameter were taken from coronary angiography with the support of clinical data of the patient. The RCA in this study was measured in three sub-divisions such as mid, distal, and proximal. Distal RCA had the least average diameter. The mid-RCA and proximal RCA had a mean diameter of 3.19 and 3.78 mm, respectively. A study reported the average diameter of RCA within the range of 2.69-3.18. The mean diameter of RCA was 2.95 mm. The mean RCA diameter of coronary heart disease patients was 2.88 mm. The study also suggested some of the cut-off values for the average diameter of RCA and LCA. For RCA, the suggested average diameter was 3.18 mm with a sensitivity of 65% and specificity of 72% [17]. If we consider this cut-off value for average RCA, we can conclude that the average diameter for proximal RCA was comparatively very high, whereas the average mid-RCA diameter was approximately the same.

In the current investigation, we observed that the difference between mid, distal, and proximal LAD was significant. The mean diameter of proximal LAD was larger than mid-LAD, and the mean diameter of mid-LAD was higher than distal LAD. We observed that the mean diameter of proximal LAD was 3.12 mm. The mean diameter of LAD was 2.26 mm in a US study conducted by Mehran et al. [17]. Another study showed that the mean diameter of proximal LAD was 3.61 mm using intravascular ultrasound and 3.36 mm using quantitative coronary analysis. However, the difference between these two measures was significant [24]. In almost all the sub-branches of LCA, we observed that the average diameter of proximal LAD and average diameter of proximal LCx were comparatively higher than mid- and distal LAD and mid- and distal LCx. Another study showed that the average diameter of LCA diameter was 3.96 mm [21].

Our findings were consistent with other research on coronary artery diameter in the Pakistani population as reported in the literature. There was a clear dominance of LCx and RCA diameter patterns. It was also speculated that coronary artery dominance can also influence the diameter of LCx and RCA [25]. Few studies yet have been published that report coronary artery diameter. To our knowledge, the literature regarding the coronary artery diameter, RCA diameter, LCA diameter, and diameter of sub-divisions of LCA in Pakistan is rare. Factors that cannot be modified for pre-mature CAD are age, gender, ethnicity, and genetic predisposition of heart diseases [26]. Also, calcium levels in CAD patients can be a potential factor for coronary artery [27]. Some studies also reported that lipid profile is also associated with early age onset of CAD. It is also responsible for major cardiovascular outcomes and mortality with coronary heart disease [28]. Hypercholesteremia and hypertriglyceridemia were found to be a potential risk factor for CAD [29]. The current study was conducted with a small number of patients, which is why the association of CAD severity with coronary artery diameter cannot be evaluated.

## Conclusions

The study aimed to observe the mean diameter of the RCA and sub-branches of the left coronary artery. The average diameter of the main coronary arteries plays an important role in the success of coronary artery bypass graft (CABG) surgery. A small mid-LAD diameter is associated with a considerably augmented risk of mortality in the hospital after CABG. Additional large-scale research is needed to investigate the effect of coronary artery width on the risk of CAD and to develop effective CAD risk-reduction strategies.
